# Photosystem II Extrinsic Proteins and Their Putative Role in Abiotic Stress Tolerance in Higher Plants

**DOI:** 10.3390/plants7040100

**Published:** 2018-11-14

**Authors:** Shina Sasi, Jelli Venkatesh, Rawya Fatohllah Daneshi, Mayank Anand Gururani

**Affiliations:** 1 Khalifa Center for Genetic Engineering and Biotechnology, United Arab Emirates University, P.O. Box 15551, Al Ain, UAE; shina_sasi@uaeu.ac.ae; 2 College of Agriculture and Life Science, Seoul National University, Gwanak-gu, Seoul 151-921, Korea; jvs15@snu.ac.kr; 3 Department of Biology, College of Science, United Arab Emirates University, P.O. Box 15551, Al Ain, UAE; 201309923@uaeu.ac.ae

**Keywords:** abiotic stress, Arabidopsis, extrinsic proteins, photosystem ii, photosynthesis, tolerance, transgenic

## Abstract

Abiotic stress remains one of the major challenges in managing and preventing crop loss. Photosystem II (PSII), being the most susceptible component of the photosynthetic machinery, has been studied in great detail over many years. However, much of the emphasis has been placed on intrinsic proteins, particularly with respect to their involvement in the repair of PSII-associated damage. PSII extrinsic proteins include PsbO, PsbP, PsbQ, and PsbR in higher plants, and these are required for oxygen evolution under physiological conditions. Changes in extrinsic protein expression have been reported to either drastically change PSII efficiency or change the PSII repair system. This review discusses the functional role of these proteins in plants and indicates potential areas of further study concerning these proteins.

## 1. Introduction

**Figure 1:**
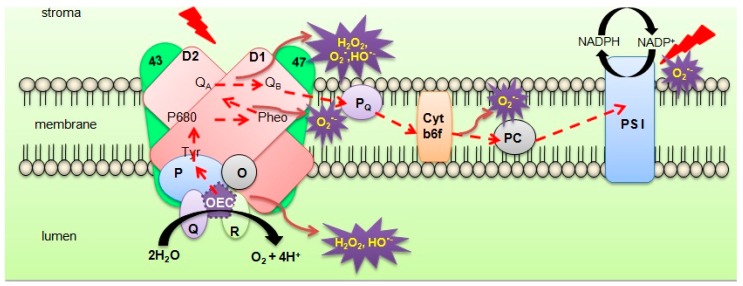
The redox chemistry of the photosynthetic electron transport chain and potential PSII target components of abiotic stress in higher plants. The diagram illustrates thylakoid membrane compartments, intrinsic and extrinsic protein complexes of the PSII reaction centre, and the sites of reactive oxygen species (ROS) molecule production. The D1 (encoded by PsbA) and D2 (encoded by PsbD) reaction centre core proteins and the 43 (CP43 encoded by PsbC) and 47 (CP47 encoded by PsbB) core antenna proteins are also shown in the diagram. The dashed red arrows represent the linear electron flow from water to NADP that takes place after water-splitting. The tyrosine (Tyr) residue is a part of the D1 protein that transfers the electrons from the 4 manganese cluster at the oxygen-evolving complex (OEC). The electrons are then transferred to P680, which is a chlorophyll molecule with its maximum absorption at 680nm. This electronis subsequently captured by a pheophytin (Pheo) molecule, which is the primary electron acceptor located near P680. Next, the electrons are transferred to the quinone acceptors QA and QB, and eventually to NADP via photosystem I (PSI) and the subsequent cellular processes (not shown in the diagram).

Photosynthesis is one of the fundamental processes that drive life on Earth. Sunlight is converted into chemical energy and is used to convert carbon dioxide, water, and minerals into oxygen and energy-rich organic compounds which are used as food or an energy source by heterotrophs [1]. A specialized multi-protein complex referred to as photosystem II (PSII) is located in the thylakoids of oxygenic organisms. Light energy absorbed by PSII generates strong oxidants that can break down water molecules. This splitting of water molecules occurs at the oxygen-evolving complex (OEC) of PSII [2,3], which is stabilized and protected by extrinsic proteins at the luminal side of PSII (Figure 1). These PSII extrinsic proteins (PsbO, PsbP, PsbQ, and PsbR) are encoded by multiple gene families in higher plants such as *Arabidopsis*, pea, tomato, and tobacco [4]. These extrinsic PSII proteins are known to be targets of stress. During the course of evolution, PSII developed the ability to use water molecules as a source of electrons, which led to oxygen accumulation in the atmosphere [5]. In cyanobacteria, algae, and plants, light is harvested in the antenna region by chlorophyll, carotenoid, and phycobilin (light-harvesting) pigments, and excitation energy is transferred to the PSII reaction centre that contains the pigment complex P_680_ [6,7]. Excitation energy causes an electron transfer to occur from P_680_ to pheophytin (pheo), which is the primary electron acceptor of PSII (Figure 1). This leads to the creation of a charge-separated state [8]. Primary charge separation is prevented by transferring electrons from a negatively charged pheo (pheo^−^) to protein-bound plastoquinones, Q_A_, and then from Q_A_ to Q_B_ (Figure 1). After transferring two equivalents, Q_B_ is converted into plastoquinol (PQH_2_) and is dispatched from PSII. Associated with these reactions, an electron from Y_Z_, a tyrosyl residue on the D1 protein from the Mn_4_CaO_5_cluster, reduces P_680_^+^(P_D1_^+^), causing the subsequent release of Y_z_ into the lumen (Figure 1). Further reduction of Y_Z_ causes acquisition of an oxidizing equivalent in Mn_4_CaO_5_, which is required for the complete oxidation of water [9,10]. The PSII protein complex contains >20 subunits and includes both extrinsic and intrinsic membrane proteins [11]. Some of the intrinsic proteins, such as PsbA (D1), PsbB (CP47), PsbC (CP43), PsbD (D2), PsbE (a subunit of cyt b_559_), PsbF (a subunit of cyt b_559_), and PsbI, are required for phototroph growth and oxygen evolution. However, the deletion of these proteins causes disruption of the PSII assembly and function [10]. The subunit structure of the intrinsic proteins has been highly conserved in cyanobacteria and higher plants and is necessary to allow oxygen evolution [6], while the extrinsic proteins have undergone a large evolutionary change.

Adverse environmental conditions such as droughts, heat, heavy metals, high-intensity light, and increased salinity result in abiotic stress, which in turn affects the total plant yield. According to an estimate, abiotic stresses account for a >50% reduction in the average yield of major crops [5,12]. Abiotic stresses that restrict the CO_2_ availability because of stomatal closure facilitate the generation of reactive oxygen species (ROS) molecules in chloroplasts. Consequently, the transpiration rate and gaseous exchange in plants are reduced, which, in turn, lead to a significantly reduced photosynthetic efficiency [13,14,15]. At a molecular level, these stresses lead to decreases in PSII activity, and over-reduction in the electron transport chain (ETC) results in the photooxidation in plants [16,17,18,19]. Plants overcome the problem of ETC reduction by converting light energy into thermal energy via non-photochemical quenching (NPQ) [3,5,20]. NPQ reduces the concentration of chlorophyll excited states (Chl*) in PSII by activation of a heat dissipation channel that facilitates NPQ as a major photo-protective response [21,22]. During high-intensity light stress, a change in chlorophyll protein distribution and molecular orientation in the thylakoid membrane is observed [23]. Abiotic stresses lead to the generation of reactive oxygen species (ROS) within the photosynthetic ETC. High ROS production under abiotic stress causes loss of crop productivity. Earlier findings have indicated that energized electrons are allocated to dioxygen (O_2_) during abiotic stress, which is used in two vital photosynthetic reactions: photorespiration and the Mehler peroxidase reaction [24,25]. Lipid peroxidation as a result of ROS production has been identified as one of the major factors that damage the PSII proteins. Abiotic stresses, particularly high-intensity light and high temperature, primarily lead to the production of ROS at the reaction centre, antenna, and in the membrane near the lipid molecules. In addition, endogenous cationic radicals are also produced at the PSII reaction centre. These primary events then cause oxidative damage and lipid peroxidation in PSII components, which eventually leads to PSII protein cleavage and aggregation [26].

D1 photo-damage is caused when light-absorbing antennae receive high-intensity light. The light-harvesting complex II (LHCII) dissipates the excess excitation energy in the form of heat through NPQ in order to counter the photo-damage. This is followed by the subsequent phosphorylation and dephosphorylating of LHCII components, in addition to the degradation of damaged D1 copies and synthesis of nascent D1 copies [5,27]. Numerous studies (reviewed in References [5,28]) have revealed many details about the influence of abiotic stress on PSII extrinsic proteins, in addition to the D1 damage repair process. In plants, PSII is separated in highly stacked membrane layers of very large thylakoid membranes [29,30]. Fristedt et al. [31] revealed that a high level of PSII phosphorylation facilitates the folding of large photosynthetic membranes which, in turn, promotes the lateral mobility of membrane proteins and ensures sustained photosynthetic activity in plants. Interestingly, Khatoon et al. [32] compared the effects of light stress on stacked and unstacked thylakoids in isolated spinach thylakoids and reported that PSII photo-inhibition was significantly higher in stacked thylakoids compared to the unstacked thylakoids. Based on their findings, the authors suggested that the unstacking of thylakoids might play a critical role in promoting the degradation of damaged D1 copies and preventing more damage to the D1 protein [32]. Recent studies have highlighted the role of micro RNA (miRNA) in the regulation of stress tolerance in higher plants. Fu et al. [33] identified novel microRNAs in maize, exhibiting relatively high abundance and significantly altered expression levels under salt stress. The authors demonstrated that some of the detected novel miRNAs may play key roles in adapting to salt stress in maize. Experimental evidence from previous reports indicates that abiotic stresses exhibit adverse effects on critical metabolic pathways, including ROS detoxification, respiration, carbon fixation, and photosynthesis, as well as respiration [14,34]. Recently, in another study,2574 mRNAs and 76 miRNAs were identified in citrus plants that showed differential expressions when exposed to salinity and drought stresses [35]. Their findings revealed that biological processes, such as those related to hormone signalling, ROS metabolism, transcription factors, and signal transduction, are involved in salinity and drought stresses [35]. Considerable progress has been made in understanding the molecular mechanism that regulates abiotic stress tolerance in plants in many plant species [36,37,38]. However, little is known about the putative roles of PSII extrinsic proteins in the abiotic stress tolerance in higher plants. In this review, we discuss the current knowledge on the major extrinsic proteins of PSII and examine their regulatory roles under abiotic stress-induced changes in the physiology of higher plants.

## 2. PsbO

**Figure 2:**
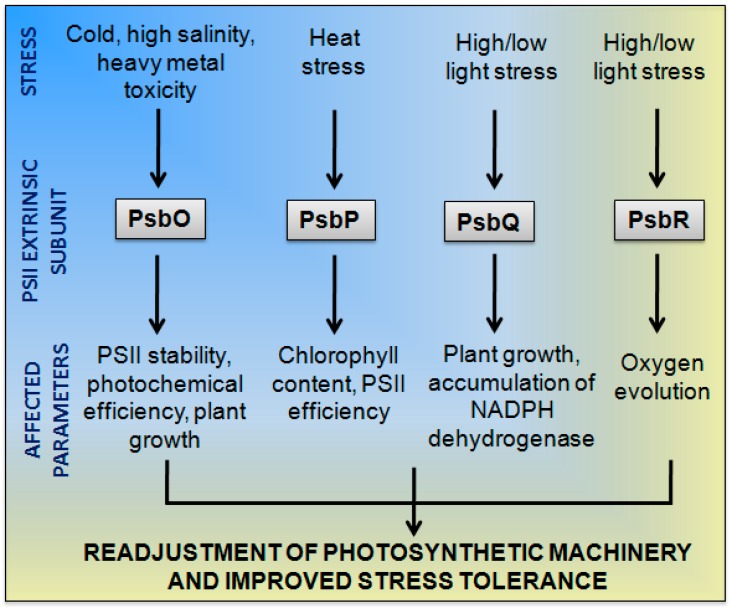
The putative involvement of extrinsic PSII subunits and the representative specific affected parameters under abiotic stress conditions in higher plants. Several studies (described in the article) have demonstrated the effect of various abiotic stress factors, such as cold, heat, high-intensity light, salinity, etc. on the extrinsic PSII proteins PsbO, PsbP, PsbQ, and PsbR in higher plants. The effects of these stresses have been observed in various photosynthetic, morphological, and physiological parameters. These studies indicate that the extrinsic PSII components might play a critical role in abiotic stress tolerance and that their functional significance in abiotic stress tolerance must be investigated.

PsbO is one of the extrinsic PSII subunits located on the luminal side of the thylakoid membrane (Figure 1). Kuwabara and Murata [39] first identified PsbO, also called the manganese-stabilizing protein (MSP), in spinach chloroplasts. PsbO, the 33 kDa protein consisting of 231–257 amino acids, is expressed in all oxygenic photosynthetic organisms [40]. PsbO is found to be highly conserved (60–80%) among higher plants and algae and around 40% conserved among lower plants and cyanobacteria [10]. Plants such as rice (*Oryza sativa*) and pea (*Pisum sativum*) have only one PsbO gene, while potato (*Solanum tuberosum*) and *Arabidopsis* (*Arabidopsis thaliana*) have two isoforms of PsbO. Analysis of the PsbO sequence demonstrated that all analysed gymnosperms seem to have only one PsbO isoform, while the majority of the analysed angiosperm species have two PsbO isoforms [41]. PsbO-1 (At5g66570) and PsbO-2(At3g50820) are two isogenes that encode PsbO in *A. thaliana* that yield two different proteins, PsbO-1 and -2. Murakami et al. [42] found a two-amino-acid difference in PsbO-1 and -2, along with functional differences and interpreted that PsbO-2 was recently derived from PsbO-1 during evolution by gene duplication. PsbO participates in calcium binding [43] and acts as a molecular-like chaperone in PSII [32,44]. In addition, PsbO has been reported to play a crucial role in two aspects: (1) protecting PSII from photo-damage and (2) oxygen-evolving complex (OEC) function [4,10,19,22]. An *Arabidopsis* PsbO-1mutant demonstrated PsbO-2 upregulation and exhibited reduced PSII activity, while those lacking PsbO-2 had a higher PSII activity. Further analysis of PsbO mutants indicated that both PsbO-1 and -2 were active in photosynthesis and PsbO-2 could be substituted for PsbO-1 in PSII activity [2]. Several other studies (listed in Table 1) have suggested that PsbO gene splay a major role in photoautotrophic growth, oxygen evolution, and PSII assembly in *Arabidopsis* mutant plants with deficient PsbO genes [45,46]. PsbO is also known to regulate D1 protein phosphorylation during PSII repair [2]. An *Arabidopsis* PsbO-1 mutant exhibited defects in the capability of PSII to utilize calcium in support of oxygen evolution [47]. Antisense reduction of PsbO proteins in *Arabidopsis* affected the functional PSII content with reduced quantum yield, but resulted in a similar photosynthetic rate as the wild-type plants [48] (Figure 2). Among the 25 core PSII protein subunits, PsbO appears to be of prime importance in the photosynthetic water-splitting process and OEC stabilization [49].

The x-ray crystallography of PSII from the cyanobacterium *Thermo synechococcus* resolved PsbO’s three-dimensional structure with a resolution of 1.9 Å [50]. The structure of the whole PSII from a higher plant was obtained by cryo-electron microscopy and cryo-electron tomography, but these techniques could not resolve the PsbO’s structure due to insufficient resolution [51]. High sequence identity of PsbO between *Thermo synechococcus* and a higher plant (about 45%) disclosed a homologous model for plant PsbO [40]. X-ray scattering data revealed that PsbO from spinach and cyanobacteria have similar structures and oxygen-evolving rates [52].

Some studies have revealed the effects of whole plant stress on the performance of the photosynthetic apparatus [53,54,55,56,57] (Table 1; Figure 2). In vitro experiments with a PsbO mutant (mutation in Trp241 in PsbO) spinach showed a high risk of photo-inhibition, accumulation of D1 and CP43, and detrimental effects on PSII binding [58]. PsbO prevents unnecessary interactions between photo-damaged D1 and CP43. The extended structure of PsbO was also found to protect D1 from ROS [32]. Pawlowicz et al. [59] reported the protective role of PsbO against photo-damage during abiotic stresses. They studied the PsbO expression pattern in forage grasses, *Festuca arundinacea*, and *F. pratensis* exposed to stresses such as cold and drought. Their results again confirmed the protective function of PsbO on the photosynthetic apparatus, in which a higher stability of PSII during the drought was observed. However, PsbO protein degradation caused the destabilization of the OEC, and it was observed that a difference in efficiency of photochemical activity and PsbO accumulation in cold-treated *F. pratensis* existed. Previously, Kosmala et al. [60] had reported that the PsbO protein in the same forage grasses was partially degraded during cold treatment and the products of its degradation were found in numerous protein spots on 2D maps. Fischer et al. [61] studied a PsbO potato mutant that exhibited altered photosynthetic machinery, resulting in physiological changes such as reducing rooting, delayed senescence, basal branching, and enhanced tuberization. Gururani et al. [19] reported that a lack of active OECs led to an increase in PSII activity for a short period, resulting in early tuberization in antisense PsbO lines. The same mutants also showed a significantly high level of tolerance against various types of abiotic stress [22]. It was observed that under high salinity, heavy metal, or drought stress conditions, the chlorophyll content, average plant height, leaf number, and tuber yield of mutant plants were much higher than the control lines. Antisense PsbO potato plants accumulated less H_2_O_2_ and activities of ROS-scavenging enzymes such as ascorbate peroxidase, catalase, superoxide dismutase, glutathione reductase, and dehydroascorbate reductase were enhanced under conditions of heavy metal, salt, and osmotic stress. Similar low levels of H_2_O_2_ accumulation in stress-tolerant plants have been reported in previous studies [14,62]. This increase in ROS-scavenging enzyme activities, along with the increased amounts of carbohydrates; plant growth regulators such as ascorbate, tocopherols, ABA, and proline; and osmolytes in PsbO mutant potato plants indicated a strong role for PsbO in abiotic stress tolerance. Interestingly, gene expression studies of intrinsic PSII proteins such as D1, D2, and CP43 revealed a significant difference in their expression levels among transgenic and control lines. Antisense PsbO potato plants [22] exhibited reduced D1 and CP43 gene expression levels under normal light conditions. In another study, the RNAi-mediated suppression of PsbO in *Arabidopsis* plants led to the loss of expression ofD1 and CP43 proteins and loss of variable fluorescence yield (Fv/Fm) [63]. Wei et al. [64] observed a PsbO upregulation along with the PsbQ, PsbP, PsbY, PsbZ, and Psb28 subunits of PSII in melatonin-treated soybean plants. Melatonin has several functions in plants, such as protecting plants from environmental stress, acting as an antioxidant, and upregulating plant growth. Melatonin-treated plants also showed an enhancement in plant growth, soybean production, fatty content, pod and seed numbers, and upregulation of the expressions of genes that are inhibited by salt and drought stress [64]. Mutations in the PsbO gene in *Arabidopsis* caused growth retardation [42]. Interestingly, reduced levels of PsbO (and thus, the reduced functioning of the OEC) were also observed in PsbS-overexpressing tobacco plants [65]. The PsbS protein in higher plants is associated with the proper dissipation of excess light energy via its regulation of NPQ. Transgenic tobacco plants with an overexpression of PsbS showed less stomatal opening, resulting in a 25% reduction in water loss that, in turn, increases the efficiency of water use [65]. Given some of the contrasting reports regarding the relationship between PsbO expression and plant growth under normal and/or stressed conditions [22,59], it is possible that it varies because of the different number of PsbO isoforms in different plant species.

## 3. PsbP

The 24 kDa PsbP protein in higher plants has been reported to play a role in optimizing Ca^2+^ and Cl^−^ availability for maintaining the Mn–Ca^2+^–Cl^-^ cluster within PSII [80]. Tomita et al. [81] reported that PsbP induces conformational changes in the Mn cluster for Ca^2+^ and Cl^-^ by interacting with PSII, and the same is supported by PsbQ. The N-terminal sequence of PsbP induces the proper conformational changes around the OEC in order to retain Ca^2+^ and Cl^-^ in PSII [81]. Enami et al. [82] suggested that PsbV/CyanoP in cyanobacteria is replaced by PsbP in higher plants in the evolutionary hierarchy. Association of cyanobacterial CyanoP with PSII function is much less in cyanobacteria [83], while in higher plants, PsbP has a dominant role in regulating and stabilizing PSII [66]. The presence of PsbP homologues along with PsbP in chloroplast thylakoid lumens have been reported [10,66,81]. The ten PsbP homologs include two PsbP-like proteins (PPL1 and 2) and eight PsbP-domain proteins (PPD, including PPDs 1–8) [67,84,85]. Studies suggest that the presence of the PsbP homologs PPL1 and 2are highly similar to that of CyanoP [10,67]. These PsbP homologs have less sequence identity (~25%) with PsbP in *Arabidopsis* [10]. It has been suggested that PsbP and PPL1 and 2 have evolved from their cyanobacterial homolog CyanoP and have undergone genetic and functional modifications that had occurred in order to generate eukaryote-like proteins [67]. PsbP homologs in *Arabidopsis* are classified into three distinct groups: (1) a group of authentic OEC proteins; (2) a second group that co-expresses with ribosomes and immunophilins and some stress-related genes; and (3) a third group with the chloroplastic NADPH dehydrogenase (NDH) complex [49]. It is suggested that PPL1 expresses with immunophilins, is involved in a stress-related group, and plays a role in the assembly of Cytb_6_f and PSII, while PPL2 is involved in the photosynthetic NDH sub-complex lumen [86].Crystalline structure analyses of PsbP from *Nicotiana tabacum* and *Spinacia oleracea* suggest that both PsbP and PsbP homologs have the same structure, called a Mog1p/PsbP-like fold, while the N- and C-terminal loop sequences linked to the central β differ among each of them [84,87]. It has been suggested that these differences conferred functional differences among the PsbP family [10,84].

PPL1and 2 of PSII has been proposed to have some function in photosynthesis under conditions of stress. Ishihara et al. [67] observed that the PPL1 mutant *Arabidopsis* plants were more sensitive to high-intensity light than wild-type plants, resulting in a photobleaching phenotype. Further, a decline in D1 accumulation was observed in these mutant plants and showed a delayed recovery of PSII activity after photo-inhibition. The results indicated that PPL1 might be a necessary component for the efficient repair of photo-damaged PSII and is essential for photoautotrophic growth [68]. On the other hand, PPL2 *Arabidopsis* mutants exhibited a defective chloroplastic NADH dehydrogenase-like (NDH) complex, which was later confirmed by Matsui et al. [69]. RNAi suppression of PsbP in *Arabidopsis* led to the loss of variable fluorescence; fluorescence quantum yield; damage of D1; D2, CP47, and CP43 accumulation(PSII core protein damage); and photoautotrophic growth [70].Similar results have shown that along with the loss of PsbQ, the accumulation of PsbO with the PSII intrinsic protein and unstable manganese cluster was observed during the analysis of RNAi-suppressed PsbP tobacco plants [66].Loss of CP47 and CP43 is mainly due to PsbP interlinking with the light-harvesting proteins CP26 and CP43, along with PsbE, PsbQ, and PsbR [88]. This clearly indicates that PsbP has several functions in PSII. Yi et al. [70] also reported that a small but detectable amount of the PsbP protein is necessary to support PSII function. Regarding this finding, a PsbP homologue was found to be sufficient to carry out all PSII functions in cyanobacteria [89]. These results confirmed that PsbP is necessary for PSII function and stability/assembly [70,90]. Roose et al. [71] reported a decrease in NDH activity along with plant developmental and phenotypical defects in PPD5 mutant *Arabidopsis* plants; this finding suggests that PPD5 is involved in strigolactone biosynthesis. PPD6 is found in the stress-responsive group, which is a putative target of thioredoxin [91].

Studies on the PPD1 mutant *Arabidopsis* plants revealed that PPD1 is essential for PSI assembly and function. PPD1 deletion caused the inability of mutant plants to grow photo-autotrophically, loss of PSI stability and integration of PsaA and PsaB into the thylakoid membrane, and defects in electron transfer from plastocyanin (PC) to the oxidised reaction centre P700^+^ [10,92]. It was found that in PsbP1 mutant *Arabidopsis* plants, the photo-autotropic growth of the mutant was affected, while the triple mutant Psbq1/psbq2/psbrlacking PsbP grew normally, indicating that PsbP1 is essential for photoautotrophic plant growth. The authors observed that at an early growth stage, small amounts of PsbP were detected, which then progressively decreased, suggesting that PsbP is required for PSII assembly in the early stages of the plant growth stages and can be dispensable in older plants [68].

A homolog of PsbP, IbPsbP, has been identified in sweet potato. Analysis of amino acid sequences confirmed that IbPsbP clusters together with other PsbP homologs in this plant. Studies of sweet potato have reported that IbPsbP is localized in chloroplasts and is upregulated during abiotic stress [72]. It was found that IbPsbP interacts with IbOr (*Ipomoea batatas* Orange protein, a protein that regulates environmental stress and the biosynthesis of carotenoids), thereby protecting it from heat denaturation. Transgenic sweet potato with the overexpression of IbOr had higher chlorophyll content and PSII efficiency during heat stress. It was observed that in these plants, IbPsbP was elevated during various types of stress, indicating that both IbOr and IbPsbP can protect plants from environmental stresses [72].

## 4. PsbQ

Both the 17kDa extrinsic proteins PsbQ and PsbP are required for the Ca^2+^ and Cl^-^ retention that is needed for PSII activity. Akabori et al. [93] and Miyao and Murata [94] first reported that PsbQ is required at low Cl^-^concentrations (<3 mM) for oxygen evolution. Both PsbQ and PsbP are responsible for multiple interactions with both PSII intrinsic and light-harvesting proteins [88]. Phylogenic studies have revealed that PsbQ in plants developed from CyanoQ in Cyanobacteria [84], and PsbQ’ is an intermediate between cyanoQ and PsbQ in *Cyanidium caldarium* (red algae) [95]. Characterization of PsbQ’ in *Cyanidioschyzon merolae* showed the nuclear regulation of PsbQ’ in influencing PSII activity, such as via dimerization regulation, partial dissociation of PsbV, and oxygen-evolving activity [96].The cyanobacterium *Synechocystis* mutant devoid of CyanoQ showed a slower growth rate and lower PSII activity with respect to the depletion of Ca^2+^ and Cl^-^; this finding suggested that CyanoQ has a similar function to PsbQ and is required for stabilizing PsbV (the response to PsbP in higher plants) binding to PSII and modulatingCa^2+^ and Cl^-^in PSII, but has different binding properties [89] than the wild-type version of the plant. Three PsbQ-like proteins (PQL) have been found in rice and *Arabidopsis* and are referred to as PsbQ-like proteins 1 (PQL-1), -2, and -3, respectively, in addition to two PsbQs in PSII [10]. These proteins are highly conserved in all higher plants, indicating their importance.

Removal of PsbQ along with PsbP caused conformational changes around the Mn cluster. Studies in spinach suggested that the N-terminal of PsbP is required for oxygen evolution and induces the protein conformation required for the retention of Ca^2+^ and Cl^-^needed for PSII activity [97]. It was also found that PsbQ can replace the N-terminal PsbP functional defect, suggesting that PsbQ plays a role in the PsbP stabilization in PSII [66]. This was later confirmed by Fourier transform infrared differential spectroscopy studies, in which PsbQ could compensate for an impaired PsbP in order to induce proper conformational changes in the Mn cluster of the water oxidation machinery [97]; this finding suggested that PsbQ interacts with PsbP in higher plants. Studies in *Salicornia veneta* (a halophyte) revealed the absence of PsbQ mRNA and its protein and had substoichiometric amounts of PsbP, leading the authors to conclude that PsbQ is not essential for photosynthesis. This may be due to the high amount of osmo-compatible solutes that allowed for the normal electron transfer from the Ca–Mn cluster [98,99]. Removal of PsbQ and PsbP caused CP29to move to the core centre of PSII, thus indicating alterations in the structural organisation of PSII [100]. 

Analysis of PQL-1 *Arabidopsis* mutants demonstrated a severe reduction in NADPH dehydrogenase levels, while a partial accumulation in the same levels was observed in PQL-2 mutants. It was also reported that PQL-3 is required for NDH accumulation and activity [73]. Observations in *Arabidopsis* mutants indicated that three PQL proteins are essential for the NADPH dehydrogenase function that regulates the plant response to both biotic and abiotic stress (Figure 2) [49,73]. However, more efforts are required to elucidate the precise functions of PQLs and PsbQs in higher plants. The studies performed in RNAi-suppressed PsbQ tobacco plants showed no phenotypic changes compared to the wild-type under normal and high-intensity light growth conditions [66]. Similar results were obtained by Yi et al. [74] after analysing the RNAi-suppressed PsbQ *Arabidopsis* plants under normal-growth light conditions. Moreover, the mutant plants showed some phenotypic changes, such as yellowing, and they died under low-light growth conditions, probably because of the loss of PSII oxygen-evolving capabilities. Further analysis revealed the loss of PSII components (D2, CP43, and CP47) and defects in electron transfer, suggesting that PsbQ is necessary for PSII function/stability and for photoautotrophic growth under low-light conditions. Allahverdiyeva et al. [68] reported that PSII–LHCII super-complexes were decreased (by up to 50%) in PsbQ and PsbR mutants. PsbQ was completely lacking in psbq1/psbq2 mutants and exhibited phenotypic changes, rapid transitions, and low levels of LHC II phosphorylation. 

## 5. PsbR

The 10kDa extrinsic protein PsbR was first identified in the spinach PSII complex [101]. Gene coding for PsbR is not found in red algae or cyanobacteria. It was suggested that PsbR is designated on the PSII luminal side, but this has not been experimentally tested. It was also suggested that PsbR is located between PsbP and PsbE [88]. The light-saturated rate of oxygen evolution is strongly reduced in the absence of PsbR, particularly in low-light-grown plants [76]. This protein is insoluble due to its hydrophobic C-terminus; hence, it easily precipitates during isolation. Although the three-dimensional structure of PsbR was predicted using I-TASSER, the PsbR’s crystal structure is not available at present [88,90].

The function of PsbR has been determined in vivo by developing antisense PsbR mutant plants. The 10 kDa protein was reduced by up to 97–99% in transgenic potato and did not show any phenotypic difference when compared with the wild-type. Analysis of these plants determined that the retardation of Q_A_ reoxidation occurred [75]. Later studies in a PsbR mutant of *Arabidopsis* reported that PsbR is required for the structural stability and proper functioning of PSII (such as maintaining its conformation and stabilizing PsbP and PsbQ binding in PSII) [76,77]. PsbR mutants exhibited PSII conformational changes, slower electron transfer, and a lower PSII activity. The authors concluded that PsbR stabilizes acceptor- and donor-side electron transfer reactions in the PSII complex. The same results, along with the reduction of the D2, PsbP, and PsbQ proteins, were obtained by Liu et al. [102]. Additionally, PsbR has been reported to stabilize PsbJ and Cytb559, which are required for PsbP and PsbQ binding and for D2 and CP47 stability [86]. PsbP, along with PsbQ and PsbR, helps to stabilize CP26 with CP43 [11,88,103]. Recently, studies have reported that the absence of PsbR causes small changes in the rate of oxygen evolution and phenotype, while depletion appears to lead to a reduced PSII activity, an impaired PSII–LHCII accumulation, and have effects on the transition states [68].

## 6. Conclusions

Upon absorption of light energy, the PSII generates strong oxidants that are capable of splitting water molecules in the OEC. The OEC is protected and stabilized by a group of extrinsic PSII proteins. Since PSII is one of the most vulnerable components of the photosynthetic machinery, it bears the brunt of the oxidative stress. Over the years, much information has been gathered about the critical components of photosynthesis. However, despite the extensive efforts that have been put into the study of PSII, a huge gap exists, especially with respect to the specific functions and properties of extrinsic PSII proteins, including their high-resolution crystal structures, their exact binding sites, and their protein interactions. It is now time to put more effort into translating this knowledge into developing photosynthetically efficient stress-tolerant crop cultivars. As mentioned earlier, a few reports have indicated the possible role of PsbO in abiotic stress tolerance in higher plants, and it is imperative to elucidate the role of other PSII extrinsic proteins using novel gene editing technologies, particularly the CRISPR/Cas9 gene editing system, which makes it possible to engineer the inherent DNA without introducing a foreign DNA sequence [104]. Ongoing efforts in our laboratory and those of others on the functional characterization of PSII extrinsic proteins using the robust CRISPR/Cas9 gene editing system along with transcriptomics, proteomics, and genomics is expected to uncover a plethora of information on their putative roles in higher plants’ abiotic stress tolerance.

## Figures and Tables

**Table 1:** The studies on PSII extrinsic proteins in various plant species.

S. No.	Plant	Stress	PSII Extrinsic Gene/Protein Mutants	Observation(s)	Reference
1	*Arabidopsis thaliana*	-	*psbO1/psbO2* mutant	PsbO1/PsbO2 are active in photosynthesis and PsbO2 substitutes PsbO1 in PSII	[2]
2	*Solanum tuberosum*	Abiotic stress (salt, heavy metal, and osmotic stress)	*psbO* mutant and *psbO* overexpressed	Mutant plant showed increased tuberization, chlorophyll content, plant height, leaf number, and increase in ROS-scavenging enzymes	[19,22]
3	*Nicotiana tabacum*	High-intensity light	PsbO	No phenotypic change	[42]
4	*Arabidopsis thaliana*	-	*psbO* mutant	Reduced quantum yield	[48]
5	*Spinacia oleracea*	-	*psbO* mutant	Photo-inhibition, accumulation of D1 and CP43, detrimental effect on PSII binding	[58]
6	*Festuca arundinacea* and *Festuca pratensis*	Cold and drought	PsbO expression pattern	Higher stability of PSII during droughts. The difference in photochemical efficiency and PsbO accumulation during cold	[59]
7	*Solanum tuberosum*	-	*psbO* mutant	Reduced rooting, delayed senescence, basal branching, and enhanced tuberization.	[61]
8	*Arabidopsis thaliana*	-	*psbO* mutant	Loss of D1, CP43, and fluorescence yield	[63]
9	*Glycine max*	Melatonin treatment		Upregulation of extrinsic and intrinsic proteins	[64]
10	*Nicotiana tabacum*		PsbS overexpressed	Reduced PsbO and increased water efficiency	[65]
11	*Nicotiana tabacum*	-	*psbP* mutant	Loss of quantum yield and PSII core proteins, loss of manganese cluster	[66]
12	*Arabidopsis thaliana*	High-intensity light	*PPL1* mutant	Photobleaching	[67]
13	*Arabidopsis thaliana*	-	*psbq1* and *psbq2* mutants	Phenotypic changes, rapid transitions, and low LHCII phosphorylation	[68]
14	*Arabidopsis thaliana*	-	*psbR* mutants	Reduced PSII activity, impaired PSII–LHCII accumulation, and effects on state transitions	[68]
15	*Arabidopsis thaliana*	-	*psbP1* mutant	Unable to grow photo-autotrophically	[68]
16	*Arabidopsis thaliana*		*PPL2* mutant	Defective NDH	[69]
17	*Arabidopsis thaliana*	-	*psbP* mutant	Loss of quantum yield and PSII core proteins	[70]
18	*Arabidopsis thaliana*	-	*PPD-5* mutant	Decreased NDH with developmental and phenotypical defects	[71]
19	*Ipomea batatas*	Environmental stress especially heat stress	IbOr	Higher chlorophyll content and PSII efficiency	[72]
20	*Arabidopsis thaliana*	Biotic and Abiotic stress	*PQL-1* and *PQL-2* mutants	Reduction in NDH accumulation	[73]
21	*Arabidopsis thaliana*	Low-light conditions	PsbQ	Exhibited phenotypic changes such as yellowing and death	[74]
22	*Solanum tuberosum*		*psbR* mutant	Retardation of Q_A_^-^ reoxidation	[75]
23	*Arabidopsis thaliana*	-	*psbR* mutant	Detrimental effects in the binding of psbP and PsbQ	[76,77]
24	*Arabidopsis thaliana*	-	*PPD-1* mutant	Unable to grow photo-autotrophically. Loss of PSI stability, loss of integrity of PsaA and PsaB incorporation into thylakoid membrane	[78,79]

## Abbreviations

CP43	chloroplast protein 43
LHCII	light-harvesting complex II
NDH	NAPDH dehydrogenase
PQL-1 and 2	PsbQ-like protein 1 and 2
Psb	Photosystem b or Photosystem II
PSI	Photosystem I
PSII	Photosystem II
QA	bound plastoquinone
ROS	reactive oxygen species
